# Synthesis of Cu-doped carbon dot/chitosan film composite as a catalyst for the colorimetric detection of hydrogen peroxide and glucose

**DOI:** 10.1007/s00604-022-05386-3

**Published:** 2022-07-19

**Authors:** Srikrishna Tummala, Rajkumar Bandi, Yen-Peng Ho

**Affiliations:** 1 Department of Chemistry, National Dong Hwa University, Hualien, 974301 Taiwan; 2 Institute of Forest Science, Kangwon National University, Chuncheon, 23431 Republic of Korea

**Keywords:** Cu-carbon dots/chitosan film, Enzyme, Peroxidase mimic, Hydrogen peroxide, Glucose, Glucose oxidase

## Abstract

The use of colloidal nanoparticles suffers from the drawbacks of potential color interference and substrate-induced aggregation. To overcome the limitations, a catalyst was developed by crosslinking Cu-doped carbon dots (Cu-CDs) with chitosan. Cu-CDs with high peroxidase activity were prepared by using a rapid microwave-assisted method. The Cu-CDs containing 6.88% of Cu had an average particle size of 2.25 nm and exhibited 9% of fluorescence quantum yield. The nanozyme/film composite was prepared by crosslinking between the amino groups of Cu-CDs and those of chitosan via a glutaraldehyde linker. A H_2_O_2_-mediated tetramethylbenzidine (TMB) oxidation reaction was use to evaluate the peroxidase activity of the film. Based on the TMB color changes, colorimetric assays were developed for the detection of H_2_O_2_ and glucose at an absorption wavelength 652 nm. Under the optimal conditions, the linear ranges for H_2_O_2_ and glucose were 0.625–40 µM and 1.9–125 µM, respectively, and the detection limits were 0.12 µM and 0.69 µM, respectively. The colorimetric assay was also applied to analyze diluted human serum samples spiked with glucose. Furthermore, this biodegradable, non-toxic, and easy-to-handle nanozyme composite could be stored for over 4 weeks without a significant decrease in activity.

**Graphical abstract:**

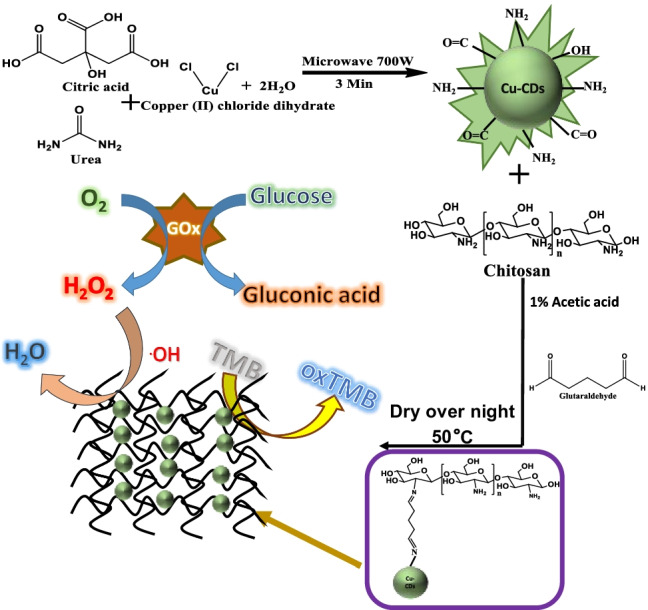

**Supplementary Information:**

The online version contains supplementary material available at 10.1007/s00604-022-05386-3.

## Introduction

The aberrant accumulation of hydrogen peroxide (H_2_O_2_) in cells leads to oxidative stress that causes cancer, aging, cardiovascular diseases, and central nervous system disorders. The abnormal level of blood sugars can lead to hyperglycemia (diabetes mellitus) or hypoglycemia. Therefore, there is a need to develop rapid, selective, and sensitive assays for the detection of H_2_O_2_ and glucose. In recent years, various techniques have been reported for the detection of H_2_O_2_ and glucose based on electrochemical, chemiluminescence, fluorescence, and colorimetric principles. Among these methods, the nanozyme-based colorimetric approach has gained significant attention because of its speed, sensitivity, accuracy, ease of operation, affordability, and potential for developing point of care sensors [1].

Enzymes have been playing an important role in many analytical applications. For instance, horseradish peroxidase (HRP) is a natural enzyme, which catalyzes the oxidation of many organic substrates in the presence of oxidizing agents such as H_2_O_2_. Therefore, HRP has been used in the detection of H_2_O_2_ by analyzing the oxidation products. Further, in the presence of oxygen, the glucose oxidase catalyzes the oxidation of glucose to yield H_2_O_2_ and gluconic acid. The amount of glucose may be indirectly determined by using the abovementioned HRP-H_2_O_2_ approach. However, natural enzymes may not be desirable for practical applications because of some limitations such as instability, difficulty in purification, and high cost. To overcome these limitations, researchers have developed stable and cost-effective enzyme mimics. For example, Fe_3_O_4_ nanoparticles were reported to show peroxidase (POD)-like activity. Several other enzyme mimics have been developed based on materials such as noble metals, carbon nanomaterials [2], metal oxide nanoparticles [3], metal complexes [4], metal–organic framework [5], and hydrogels [6]. Among the reported materials, chitosan is favorable in developing catalyst-based sensors because of its low production cost, availability, and nontoxicity.

Various nanomaterials including silver, gold, and carbon dots have been incorporated into the chitosan films [7–9]. Notably, the carbon dots possess features such as high stability, tunable optical properties, nontoxicity, good biocompatibility, and ease of synthesis as well as functionalization [10]. Because of these peculiar properties, carbon dots have been applied to the fields of sensors, bio-imaging, photocatalysis, and artificial enzymes [11]. Doping of metal atoms into the carbon dots may enhance the optical properties, change the intrinsic electronic characteristics, and create an active site with novel functions. Moreover, the combination of carbon dots with metals improves the catalytic activity of carbon dots because of the synergism between the metal atom and the carbon lattice [11, 12].

Most of the nanomaterial-based artificial enzymes (nanozymes) used for glucose detection were focused on colloidal systems. The colloidal systems tend to cause particle aggregation when analyzing biological samples, and the formation of protein corona on the surface of nanoparticles [13] may cause inconsistency in the quantification of biomolecules. To overcome the problems, we have developed a Cu-CD/chitosan film composite that may easily avoid the interference of complex biological matrices during analysis. Up to date, very few researchers have developed nanozymes embedded in films. Karim et al. prepared Ag nanoparticles embedded in cotton fabric, for the detection of glucose [14]. By selecting cotton as a template, it could increase the number of active sites and shorten the analysis time. It is useful to develop such type of nanozyme for multiple applications with improved sensitivity.

In this work, we developed a Cu-CD/chitosan film composite with high POD-like activity. Compared with the pure chitosan film, the Cu-CD-incorporated chitosan film owned better peroxidase activities. Colorimetric assays were developed for the detection of H_2_O_2_ and glucose using the proposed Cu-CD/chitosan film as a sensing platform. The dynamic range and limit of detection for the assays were investigated.

## Experimental

### Materials and instruments

Glucose, sucrose, fructose, 3,3′,5,5′-tetramethylbenzidine, glucose oxidase from *Aspergillus niger*, glutaraldehyde solution grade II 25% in H_2_O, copper (II) chloride dihydrate (CuCl_2_·2H_2_O), hydrogen peroxidase (30%), and urea were purchased from Sigma-Aldrich (MA, USA). Acetic acid (99.8%) was purchased from Honeywell Fluka (Germany). Citric acid anhydrous was purchased from J. T. Baker (NJ, USA). Chitosan (50,000–150,000 Da) was purchased from Biosynth Carbosynth (UK). Milli-Q water of 18.2 MΩ cm was produced using the Millipore water purification system. All the chemicals used in these assays were of analytical grade.

The UV–visible absorbance and fluorescence spectra were recorded on a Spectra Max® ID3 multimode microplate reader (Molecular Devices, CA, USA). X-ray photoelectron spectroscopy was performed on a Thermo scientific K-Alpha X-ray spectrometer (MA, USA). The high-resolution transmission electron microscopy images were acquired using a JEM-2001F instrument (JOEL, Japan). The Cu-CD/chitosan film morphology was recorded using a field emission scanning electron microscope (FE-SEM, JSM-7500F, JOEL, Japan). The Fourier-Transform Infrared (FT-IR) spectra were recorded on a Spectrum One spectrometer (PerkinElmer, MA, USA).

### Synthesis of Cu-CD cross-linked chitosan film

Cu-CDs were prepared by a simple one-step microwave-assisted approach as described in the Supporting Information. Chitosan (100 mg) was dissolved in 10 mL of 1% acetic acid solution with continuous stirring at room temperature for 2 h. After various amounts of Cu-CDs were added to the chitosan solution, the mixture was stirred vigorously for 1 h and sonicated for 5 min to remove air bubbles. Then, 100 µL of 25% glutaraldehyde (amine cross-linking agent) was added to the mixed solution. After 15 min of reaction, the solution was transferred to a petri dish (100 mm × 20 mm) and allowed to dry at 60 °C for 24 h. For further analysis, the formed film was cut into circular sheets by using a paper punching machine.

### Enzyme kinetics of Cu-CD/chitosan films

The Michaelis–Menten equation was applied to the kinetic analysis of the Cu-CD/chitosan-catalyzed oxidation reaction of TMB with H_2_O_2_ by fixing the concentration of either substrate. The reaction was monitored using UV–vis spectrometry. Initial reaction velocities (rates) were determined based on the absorption (at 652 nm) vs reaction time plot for the oxidized TMB product. The kinetic plots of initial rates were obtained as a function of varying concentrations of TMB or H_2_O_2_ while keeping the other component at a constant concentration. The Michaelis–Menten parameters, *K*_M_ and *V*_m_, were estimated by using the Lineweaver–Burk plot, 1/V = (*K*_M_/*V*_m_) (1/[*S*]) + (1/*V*_m_), where *V* is the initial reaction rate, *V*_m_ is the maximum reaction rate, [*S*] is the substrate concentration, and *K*_M_ is the Michaelis constant.

### Colorimetric assay for the detection of H_2_O_2_

For the analysis of H_2_O_2_, 100 µL of 6 mM TMB and 100 µL of various concentrations of H_2_O_2_ were added to 800 µL of acetate buffer (pH 4). The reaction solution was mixed with a sheet of Cu-CD/chitosan film and incubated at 37 °C for 1 h. Then, the absorbance of the solution was recorded at various concentrations of H_2_O_2_ by using UV–vis spectroscopy with an excitation wavelength of 652 nm.

### Colorimetric assay for detection of glucose

To analyze glucose, 10 µL of 10 mg/mL glucose oxidase (GOx) and 50 µL of various concentrations of glucose were added to 40 µL of acetate buffer and the solutions were incubated at 37 °C for 2 h. After the incubation, 100 µL of TMB and 800 µL of acetate buffer were added to the reaction solutions. Then, the reaction mixtures were mixed with a sheet of Cu-CD/chitosan film using a vortex mixer and incubated at 37 °C for 1 h. The absorbance of the solutions was recorded by using UV–vis spectroscopy at a wavelength of 652 nm. Human serum was obtained from male AB plasma (Sigma, product no: H4522). The serum was diluted 100-fold with an acetate buffer to minimize the glucose concentration in the real sample. Glucose was directly spiked into the serum solution at various concentrations (10, 20, 30 µM). Further, 10 µL of 10 mg/mL glucose oxidase and 50 µL of various concentrations (10, 20, 30 µM) of glucose solution were added to 40 µL of acetate buffer, and the solutions were incubated at 37 °C for 2 h prior to the glucose analysis. A diluted (reference) serum sample was treated the same way prior to the glucose analysis. The UV absorbance of the spiked samples at 652 nm was measured using the reference sample as a blank.

## Results and discussion

### Synthesis of Cu-CDs and Cu-CD/chitosan

**Scheme 1:**
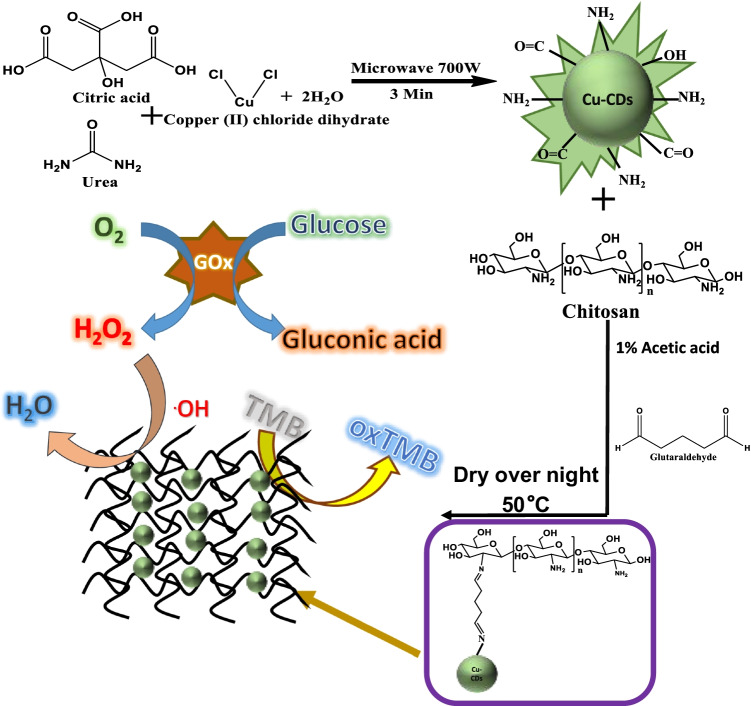
Schematic representation for the synthesis of Cu-CD/chitosan film and the detection of H_2_O_2_ and glucose

In this work, we report a simple microwave pyrolysis approach for the synthesis of Cu-CDs using citric acid, urea, and copper (II) chloride dihydrate as precursors. The synthesized Cu-CDs exhibited a quantum yield of 9 ± 2.41% (*n* = 3, Figure S1). Although the domestic microwave used in the approach is not a device for a stable power output, the quantum yield for the prepared Cu-CDs is quite reproducible. Scheme 1 illustrates the synthesis of Cu-CD/chitosan film and the detection of H_2_O_2_ and glucose using the synthesized composite. The Cu-CD/chitosan film was used as a peroxidase mimic. In the presence of peroxidase, TMB will be oxidized by hydrogen peroxide and turned into blue color, which can be monitored at a maximum absorption wavelength of 652 nm. In order to obtain Cu-CDs with the best POD activity, we have optimized the amount of copper precursor. As shown in Figure S2a, increasing the amount of copper precursor from 50 to 200 mg caused increasing absorbance of the oxidized TMB product at 652 nm, reflecting a higher catalysis activity at a higher copper content. When the amount of copper precursor was increased to 300 mg, the reaction showed an excessive charring phenomenon and the synthesized particles did not exhibit fluorescence. Therefore, 200 mg of copper chloride (II) dihydrate was used in the final synthesis. The synthesized Cu-CDs were cross-linked with chitosan through glutaraldehyde. The effect of the Cu-CD amount on the POD activity of Cu-CD/chitosan film was also studied. As shown in Figure S2b, the absorbance of the oxidized TMB product was increased with increasing Cu-CD content from 0 to 3% and leveled off when the Cu-CD content was greater than 3%. The chitosan alone (0% Cu-CDs) also exhibited POD activity, which was consistent with the previous report [15].

### Characterization of Cu-CDs

**Fig. 1:**
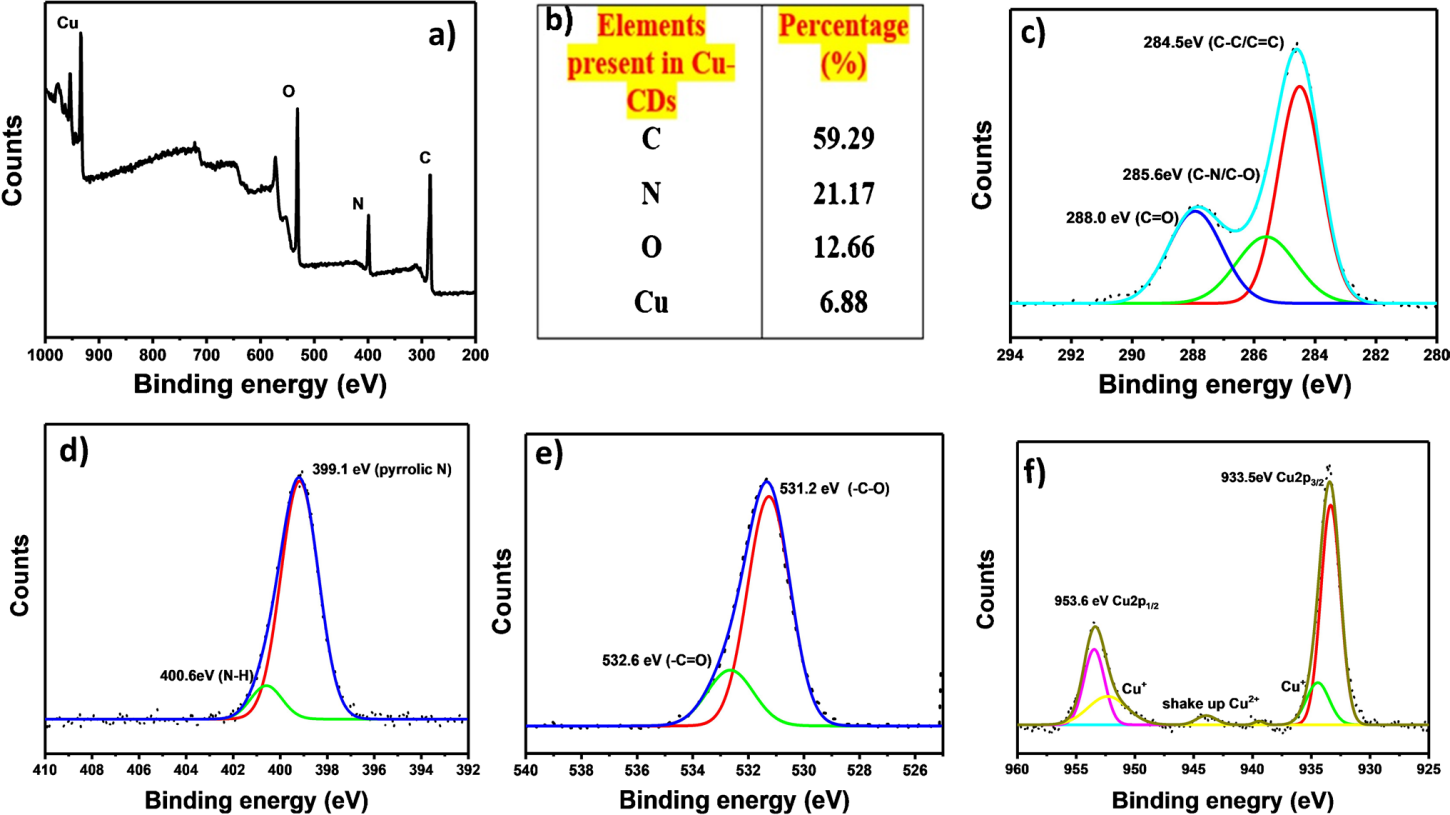
**a** XPS full scan spectrum of Cu-CDs. **b** Elemental composition of CDs. High-resolution scans of **c** C1s, **d** N1s, **e** O1s, and **f** Cu2p

X-ray photoelectron spectroscopy (XPS) was employed to investigate the chemical composition and surface states of Cu-CDs. Figure 1a reveals a full scan spectrum with four major peaks at 284 eV, 400 eV, 531 eV, and 933 eV, which are attributed to C1s, N1s, O1s, and Cu2p electrons, respectively. The Cu-CDs consist of 59.29% C, 21.17% O, 12.66% N, and 6.88% Cu (Fig. 1b). The data indicate that the copper was successfully doped into the carbon dots. High-resolution XPS spectra were background subtracted and fitted by using the Gaussian–Lorentzian function. The high-resolution C1s spectrum (Fig. 1c) exhibits three main peaks corresponding to C–C/C = C (284.5 eV), C–N/C–O (285.6 eV), and C = O (288.0 eV). The high-resolution N1s spectrum (Fig. 1d) reveals the presence of amino N (399.1 eV), and pyrrolic N (400.6 eV). The high-resolution O1s spectrum (Fig. 1e) shows two peaks associated with –C–O (531.2 eV) and –C = O (532.6 eV). Figure 1f displays a high-resolution Cu2p spectrum which has two major peaks corresponding to Cu2p_3/2_ (933.4 eV) and Cu2p_1/2_ (953.4 eV) and two minor peaks associated with Cu^+^ ion [16].

The morphology of the as-prepared Cu-CDs was investigated by using TEM. The TEM image of Cu-CDs shown in Figure S3a suggests that the particles are mostly in a spherical shape and well dispersed. The Cu-CDs have a narrow size distribution between 1.2 and 4 nm with an average size of 2.25 nm (± 0.65). The X-ray diffraction pattern and FT-IR spectra of Cu-CDs are shown in Figure S3b and S3c, respectively.

### Characterization of Cu-CD/chitosan films

The thickness and morphology of Cu-CD/chitosan films were measured by using FE-SEM. Figure S3d illustrates the cross-sectional image of the Cu-CD/chitosan film, which has a thickness of 13.48 ± 4.39 µm. The SEM image and FT-IR spectra of Cu-CD/chitosan films are displayed in Figure S3e and S3f, respectively. Detailed interpretation is described in the Supporting Information. The diameter of the circular Cu-CD/chitosan film is around 0.6 cm as shown in Figure S4.

### Optical properties of Cu-CDs and Cu-CD/chitosan films

**Fig 2:**
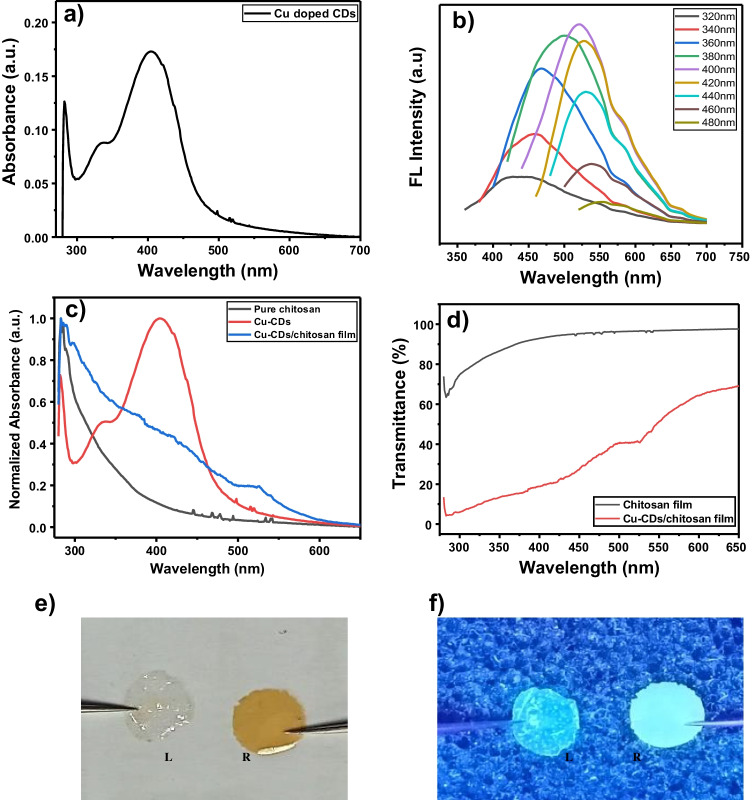
**a** Absorption spectrum of Cu-CDs. b Fluorescence spectra of Cu-CDs at various excitation wavelengths. **c** Normalized absorption spectra of chitosan film, Cu-CDs, and Cu-CD/chitosan film. **d** Transmittance spectra of chitosan film and Cu-CD/chitosan film. Digital photographs of chitosan film (L) and Cu-CD/chitosan film (R) **e** under daylight and **f** under 365 nm UV light

Figure 2a shows a UV–visible absorption spectrum of Cu-CDs in water. The peaks at 405 nm and 334 nm are attributed to the n-π* transition of conjugated C = N/C = O bonds (surface states) and the peak at 280 nm is assigned to the π-π* transition of the graphitic sp^2^ domain. The excitation spectra of Cu-CDs are shown in Fig. 2b. The Cu-CDs exhibit excitation-dependent emission characteristics with a maximum emission at 505 nm (excitation irradiation at 400 nm). The tunable emission behavior might be attributable to the surface states associated with C = O/C–N/NH_2_ of Cu-CDs.

In addition to IR spectra, UV–visible spectra were used to confirm the successful preparation of Cu-CD/chitosan film by comparing the spectra between the pure chitosan and the Cu-CD/chitosan film. As shown in Fig. 2c, the normalized UV–visible spectrum of pure chitosan has one major peak at around 285 nm corresponding to residual amide group from the chitin precursor. The Cu-CD/chitosan film contains one major peak at 283 nm which might be attributed to the n-π^*^ transition of chitosan and a small hump around 410 nm might be arising from the n-π^*^ transition of Cu-CDs. The UV–visible transmittance of the chitosan film (black curve) and Cu-CD/chitosan film (red curve) are shown in Fig. 2d. The transmittance of chitosan film is above 62% in the UV region and 90% in the visible region. With the incorporation of Cu-CDs into the chitosan film, the transmittance was expectedly decreased to 4% in the UV region and decreased to 23% in the visible region. Figure 2e and f show the photos of chitosan film and Cu-CD/chitosan film under daylight and 365 nm UV irradiation. Under the daylight condition, the chitosan film appears transparent whereas the Cu-CD/chitosan film is brown. Under the UV irradiation, the Cu-CD/chitosan film presents a uniform fluorescence, indicating that the Cu-CDs are dispersed evenly in the film.

### Peroxidase-mimicking activity of Cu-CD/chitosan film

**Fig. 3:**
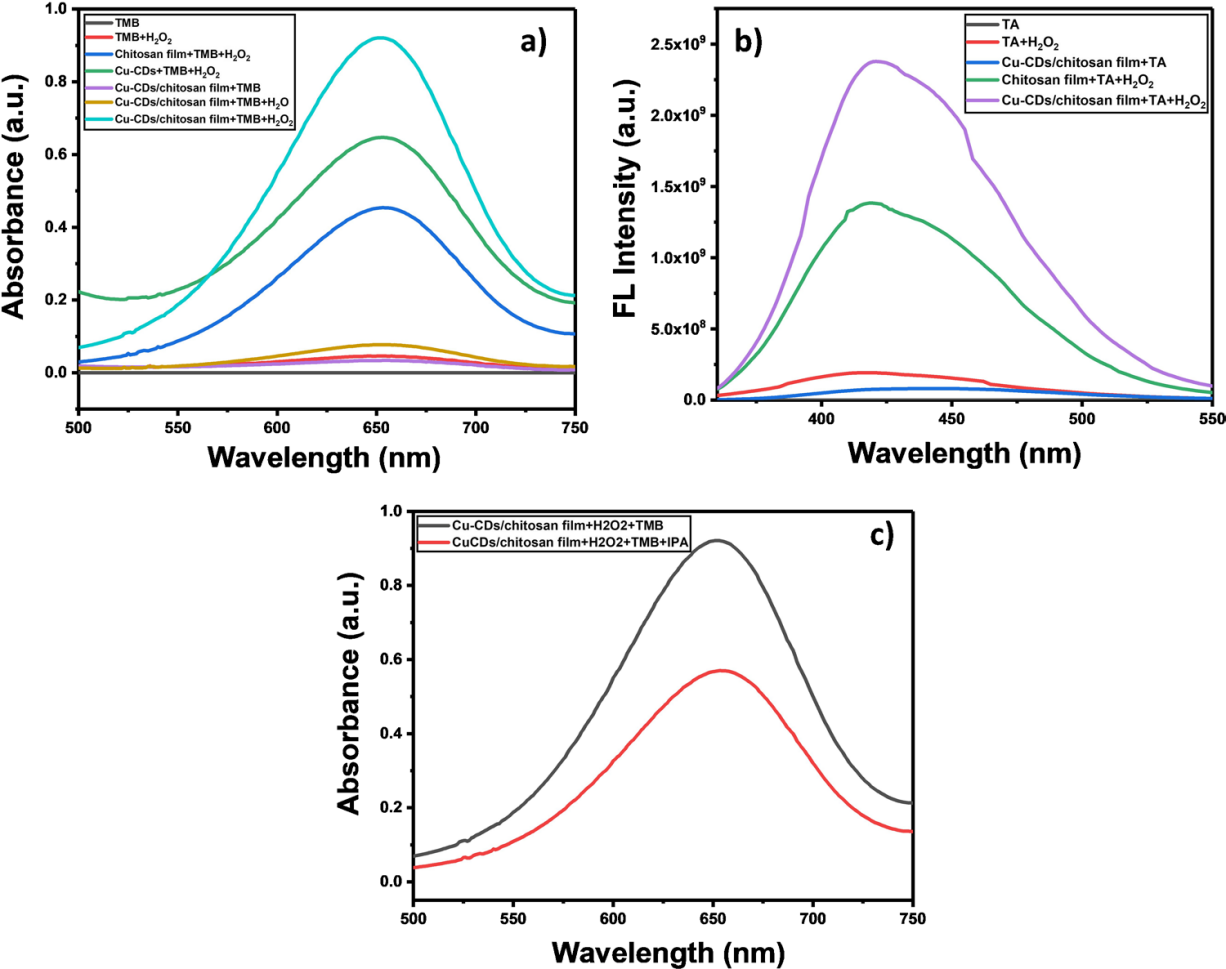
**a** Absorption spectra obtained from the catalytic reaction of Cu-CD/chitosan film, TMB, and H_2_O_2_ as well as other mixtures. **b** Fluorescence spectra of several reaction mixtures. **c** UV–vis absorbance of oxidized TMB for the Cu-CD/chitosan + TMB + H_2_O_2_ system with or without the hydroxyl radical scavenger, isopropanol

Although copper nanoparticles are known to catalyze reactions due to their wide range of accessible oxidation states, the main limitation is their ease of oxidation under air [17]. Doping copper into carbon dots may stabilize copper [18] and combining Cu-CDs with chitosan film is to prevent particle aggregation during sample analysis. We evaluated the POD activity of Cu-CD/chitosan film using TMB as a chromogenic substrate and hydrogen peroxide as an oxidizing agent. Figure 3a displays the UV–visible spectra of TMB, TMB + H_2_O_2_, chitosan film + TMB + H_2_O_2_, Cu-CD/chitosan film + TMB, Cu-CD/chitosan film + TMB + H_2_O, and Cu-CD/chitosan film + TMB + H_2_O_2_ in acetate buffer (pH 4) after 60 min of incubation. The chitosan film + TMB + H_2_O_2_ and Cu-CD/chitosan film + TMB + H_2_O_2_ system have an absorbance peak at 652 nm, which indicates that both chitosan film and Cu-CD/chitosan film may catalyze the oxidation of TMB in the presence of H_2_O_2_. The absorbance of Cu-CD/chitosan film + TMB + H_2_O_2_ is much greater than that of chitosan film + TMB + H_2_O_2_. The incorporation Cu-CDs in chitosan film significantly enhanced the catalytic activity of chitosan film. All of the other mixtures in control experiments, including Cu-CD/chitosan film + TMB, Cu-CD/chitosan film + TMB + H_2_O, and TMB + H_2_O_2_, only showed low background absorbance at 652 nm.

### Catalytic mechanism of Cu-CD/chitosan films

The POD activity of the Cu-CD/chitosan films might be derived from the catalytic decomposition of H_2_O_2_ into hydroxyl radical (^·^OH) [19]. The formation of ^·^OH may be monitored by using terephthalic acid (TA) as a fluorescent probe. The short-lived ^·^OH produced from the reaction of H_2_O_2_ with the Cu-CD/chitosan film may easily react with TA to form a fluorescent 2-hydroxylterephthalic acid. As shown in Fig. 3b, the reaction mixture of Cu-CD/chitosan film, TA, and H_2_O_2_ as well as the mixture of chitosan film, TA, and H_2_O_2_ exhibited strong fluorescence at 425 nm (under 319 nm excitation). In contrast, weak fluorescence was observed for solutions of TA, TA + H_2_O_2_, and Cu-CD/chitosan film + TA. The results proved that ^·^OH was indeed produced when H_2_O_2_ was mixed with the Cu-CD/chitosan film. We further conducted a ^·^OH scavenging experiment by using isopropanol (IPA) as a ^·^OH scavenger. As shown in Fig. 3c, the absorbance of oxidized TMB for the Cu-CD/chitosan + TMB + H_2_O_2_ system is decreased when IPA was added to the system. These results confirmed that the hydroxyl radicals were generated from H_2_O_2_ via enzymatic catalysis by the Cu-CD/chitosan film and, then, the radicals oxidized the TMB.

### Kinetic studies of the enzyme activity of Cu-CD/chitosan films

**Fig. 4:**
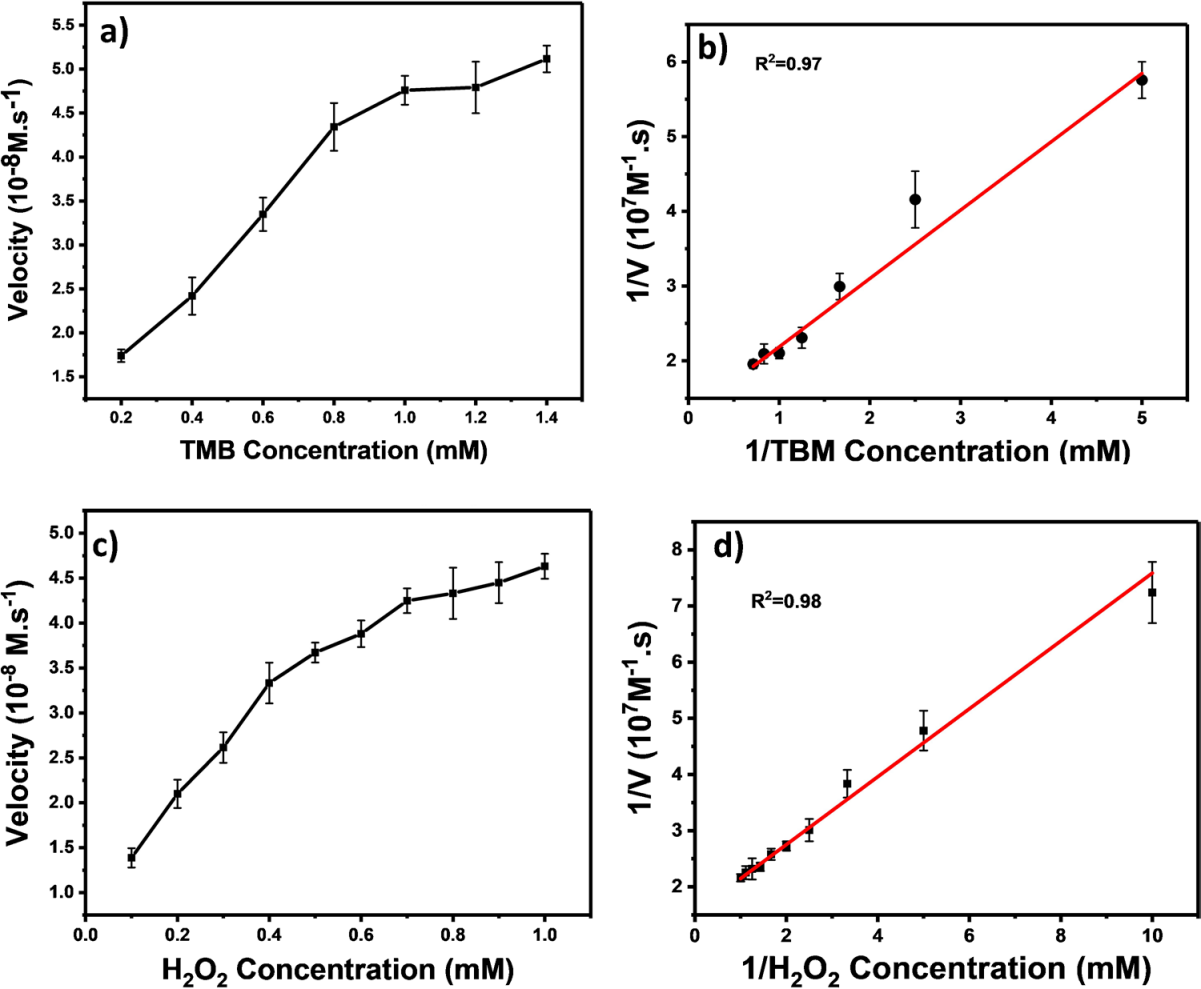
Kinetic study for the Cu-CD/chitosan + TMB + H_2_O_2_ system: a initial rate vs concentration plot and b the Lineweaver–Burk plot for the TMB substrate; c initial rate vs concentration plot and d the Lineweaver–Burk plot for the H_2_O_2_ substrate. Error bars represent the standard deviation of three independent measurements

The enzyme kinetics of Cu-CD/chitosan film was studied according to the Michaelis–Menten model. The initial rate of reaction was measured at various concentrations of either H_2_O_2_ or TMB while keeping the other substrate at a fixed concentration [20]. The maximum rate (*V*_m_) of the reaction and the Michaelis constant (*K*_M_) were determined through Lineweaver–Burk plots. Figures 4a and c display the kinetic plots of initial rate of reaction versus the concentration of H_2_O_2_ and TMB, respectively. The *K*_M_ value is useful in estimating the binding affinity of the enzyme for the substrate. The smaller *K*_M_ value represents a higher binding affinity to the substrate. From the Lineweaver–Burk plots shown in Fig. 4b and d, the obtained *V*_m_/*K*_M_ values for both TMB and H_2_O_2_ substrates were 7.8 × 10^−7^ Ms^−1^/0.71 mM and 6.4 × 10^−7^ Ms^−1^/0.38 mM, respectively. The *K*_M_ value of Cu-CD/chitosan film with H_2_O_2_ is less than that with TMB, suggesting that the prepared Cu-CD/chitosan film had a higher affinity towards H_2_O_2_ than TMB. The literature *K*_M_ and *V*_m_ values of CuNPs, CuNPs/g-C_3_N_4_, and HRP [21–23] are listed in Table S1. The *K*_M_ of Cu-CD/chitosan film with the H_2_O_2_ substrate has the lowest value of 0.38 mM among the four enzymes. The value is 10 times less than the HRP *K*_M_ value.

### Optimization of POD activity of Cu-CD/chitosan films

In order to enhance the POD-like activity of Cu-CD/chitosan biofilm, we optimized various experimental conditions including temperature, pH, the concentration of the substrate (TMB), and incubation time. The catalytic activity of Cu-CD/chitosan film was found to increase with the increase of reaction temperature from 20 to 37 °C (Figure S5a) and decrease when the reaction temperature was increased to 55 °C. The optimum temperature was at 37 °C. Figure S5b depicts that the absorbance of the catalytic product (oxidized TMB) was maximum at pH 4. This is consistent with the literature report that the POD catalytic efficiency was found to be higher under acidic conditions [20]. The absorbance of the catalytic product increased with the increasing incubation time (Figure S5c) and 60 min was enough to reach the maximum absorbance. The TMB concentration also affects the catalytic activity. The Cu-CD/chitosan activity increased when the TMB concentration was increased from 0.2 to 0.6 mM and leveled off when the TMB concentration was higher than 0.6 mM (Figure S5d). The overall optimum catalytic conditions were set at 37 °C, pH 4, 60 min of reaction time, and 6 mM of TMB concentration.

### Colorimetric assay for H_2_O_2_ detection

**Fig. 5:**
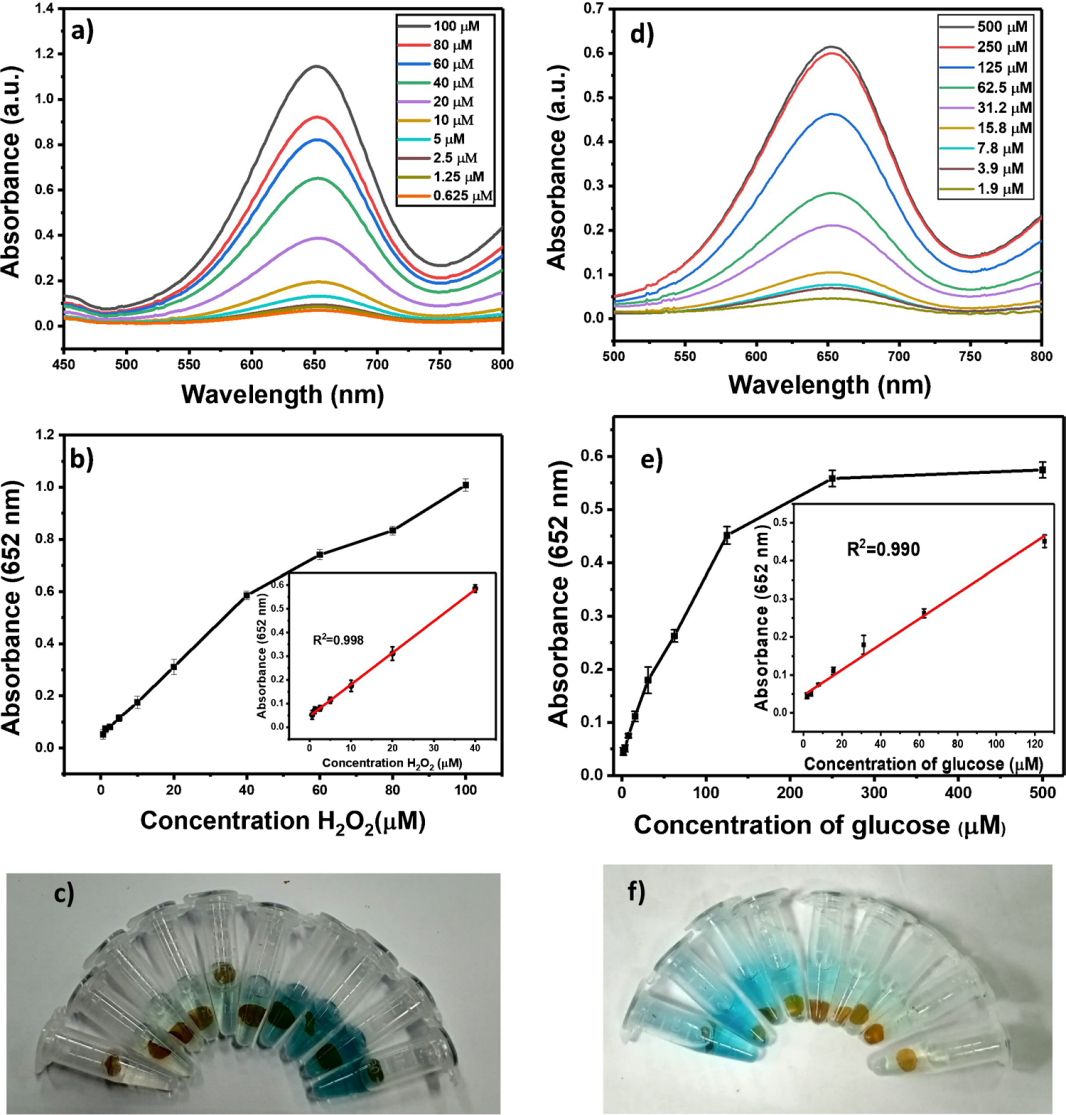
**a** UV–visible spectra of the reaction mixture of TMB/H_2_O_2_/Cu-CD/chitosan film at various concentrations of H_2_O_2_. **b** Absorbance at 652 nm vs H_2_O_2_ concentration. The inset plot shows a linear relationship (*R*^2^ = 0.998) between the mixture absorbance and H_2_O_2_ concentration. **c** Photograph of the reaction mixture of TMB/H_2_O_2_/Cu-CD/chitosan film at various concentrations of H_2_O_2_ (concentration increased from left to right). **d** UV–visible spectra obtained after the glucose oxidation at various glucose concentrations were monitored using the H_2_O_2_ detection assay. **e** Absorbance at 652 nm vs glucose concentration. The inset plot shows a linear relationship (*R*^2^ = 0.990) between the mixture absorbance and glucose concentration. **f** Photograph for the reaction mixture of glucose/GOx/TMB/Cu-CD/chitosan film at various concentrations of glucose (concentration increased from right to left)

The TMB colorimetric assay was applied to determine the sensitivity of Cu-CD/chitosan films towards the detection of H_2_O_2_. The absorption spectra of the reaction mixture of TMB/H_2_O_2_/catalyst were recorded using UV–visible spectrometry. As shown in Fig. 5a, the absorbance of the mixture increased with the increasing concentration of H_2_O_2_. Figure 5b displays the peak absorbance at 652 nm vs H_2_O_2_ concentration. The inset plot shows a good linear correlation (*R*^2^ = 0.998) between the mixture absorbance and H_2_O_2_ concentration ranging from 0.625 to 40 µM. We could visually observe the color changes of intensity with the changing H_2_O_2_ concentrations in the system as shown in Fig. 5c. The detection limit of H_2_O_2_ was 0.12 µM. The value was determined using the equation: detection limit = 3σ/m (where σ is the standard deviation of five measurements of blank signals and *m* is the slope of the linear regression).

### Colorimetric assay for glucose detection

We further applied the Cu-CD/chitosan film system to the detection of glucose. The glucose was oxidized by glucose oxidase. Because H_2_O_2_ is a byproduct of glucose oxidation [24], the developed Cu-CD/chitosan film probe may quantify the glucose concentration through the H_2_O_2_ detection assay. In the H_2_O_2_ detection assay, the absorbance of the oxidized TMB substrate was monitored. To improve the detection limit of glucose, we optimized the reaction parameters such as the concentration of glucose oxidase and incubation time. The UV absorbance of the oxidized TMB was recorded at 652 nm for the reactions carried out at various concentrations of GOx and incubation times. As shown in Figure S6a, the absorbance reached a maximum at a concentration of 10 mg/mL GOx. When the concentration of GOx was further increased, the absorbance was decreased because a large amount of converted H_2_O_2_ may lead to over oxidation of TMB. The over oxidation caused the color change of the oxidized product from blue to yellow and decreased the absorbance at 652 nm. The glucose was completely oxidized within 90 min according to the incubation time study (Figure S6b). The absorption spectra of oxidized TMB at various concentrations of glucose are displayed in Fig. 5d. A good linear correlation curve (*R*^2^ = 0.990) was obtained for the glucose analyte with a concentration ranging from 1.9 to 125 µM (Fig. 5e). The limit of detection for glucose was 0.69 µM, which was estimated according to 3σ/m. The color changes of the system solution with increasing glucose concentrations could be observed as shown in Fig. 5f. Table 1 compares the concentration range and limit of detection between the proposed Cu-CD/chitosan probe and other reported methods [14, 22, 25–29]. The silver nanoparticles on graphene quantum dots provide the best limit of detection for glucose and H_2_O_2_ [29]. However, the particles were not applied to the analysis of real sample matrices. Chitosan-modified gold nanoparticles have wide linear ranges but the limit of detection is moderate [26]. The Ag nanoparticles embedded in cotton fabric was successfully applied to the detection of glucose in urine [14]. The concept in designing the material is most similar to that in our approach. Our approach has a much better detection limit than that using the Ag-cotton fabric approach. This type of approach circumvents the problem of particle aggregation that occurred in most particle-based sensing platforms. Further, although our Cu-CD/chitosan film is relatively complex, the material is relatively easy to recover from the samples and be reused.

**Table 1:** Comparison of H_2_O_2_ and glucose detection assays between the Cu-CD/chitosan film approach and other reported methods

Nanozyme	H_2_O_2_/glucose linear range (µM)	H_2_O_2_/glucose LOD (µM)	Reference
AuNPs@C.CNF	0.5–30/1–60	0.3/0.67	[28]
GQDs/AgNPs	0.1–100/0.5–400	0.033/0.17	[29]
CuNPs/g-C_3_N_4_	0.1–2/1–100	0.032/0.37	[22]
g-C_3_N_4_	5–30/5–100	0.9/1	[25]
Chitosan-Au	1–5000/6–140	0.6/3	[26]
MoS_2_ nanosheets	5–100/5–150	1.5/1.2	[27]
Ag@fabric	–/100–2000	–/80	[14]
Cu-CD/chitosan film	0.625–40/1.9–125	0.12/0.69	Present method

### Selectivity and stability

**Fig. 6:**
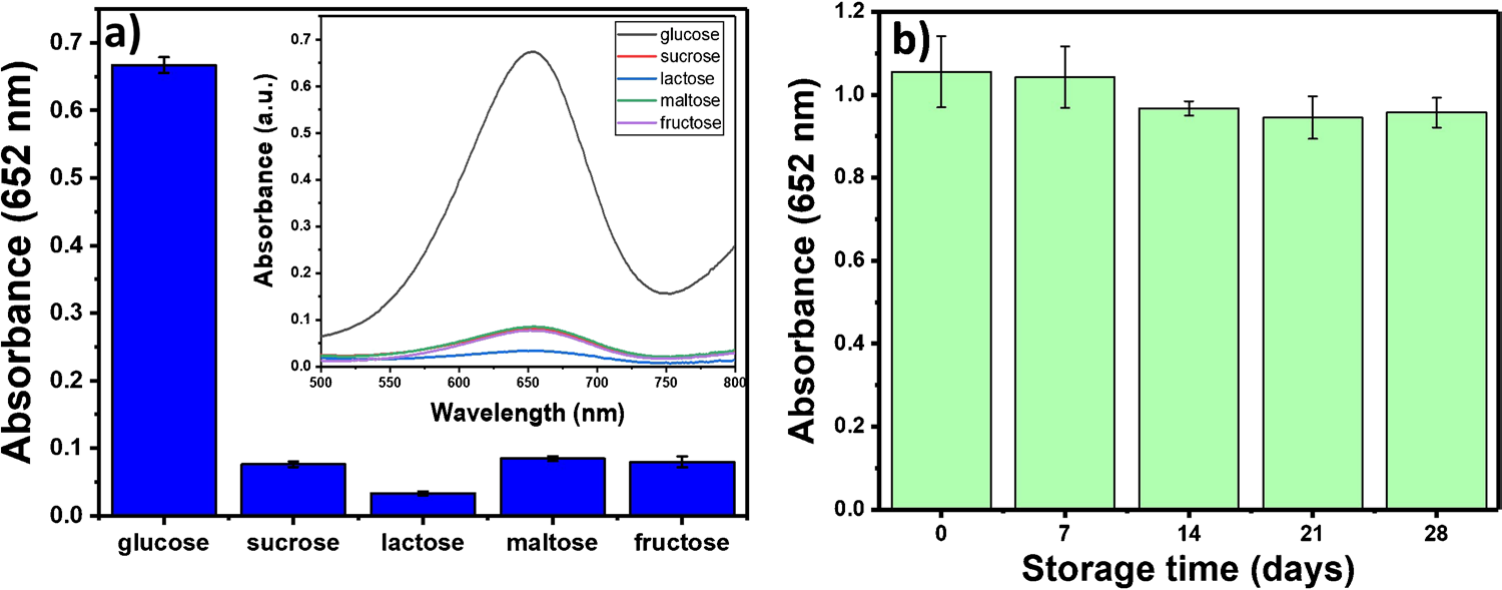
**a** Absorbance of oxidized TMB for the H_2_O_2_ detection probe towards various targets including glucose, sucrose maltose, lactose, and fructose. **b** Absorbance of oxidized TMB when the catalyst was used after being stored in a refrigerator for a period of time (*n* = 3)

To investigate the selectivity of the proposed probe towards glucose, control experiments were conducted on different analytes including glucose, sucrose, maltose, lactose, and fructose. Figure 6a illustrates the absorbance of oxidized TMB for the analytes. There is no significant absorbance for sugars except glucose. When glucose was mixed with other sugars, the absorbance remained steady (Figure S7). The results indicate that the developed system is very selective towards glucose.

The as-prepared Cu-CD/chitosan film had good stability over a long period of storage time. Figure 6b shows the absorbance of the oxidized TMB product when the Cu-CD/chitosan film was used after being stored in a refrigerator for a period of time. The absorbance remained more than 85% of the original value after 30 days of storage, suggesting stable POD activities of the Cu-CD/chitosan film. One major limitation of the present approach is the somewhat long catalytic reaction time (1 h).

The glucose detection assay was applied to analyze the glucose spiked in human serum at various concentrations. The concentration of spiked glucose was determined using the established calibration curve shown in Fig. 5e. Because the serum contains intrinsic glucose, the serum samples were diluted 100 times prior to analysis. Table S2 lists both the theoretical and experimental concentrations of glucose. The relative standard deviations of the observed values are less than 7%.

## Conclusions

We have developed a free-standing Cu-CD/chitosan film with a good POD activity. The Cu-CDs were prepared by simple microwave pyrolysis. The as-prepared Cu-CDs were incorporated into chitosan films via a glutaraldehyde crosslinking reaction. The POD activities of the pure chitosan film and Cu-CD/chitosan film were explored by the catalytic oxidation of TMB in the presence of H_2_O_2_. The POD activity was improved when the Cu-CDs were incorporated into the chitosan film. Assays were established under optimal conditions for the detection of H_2_O_2_ and glucose with good linear ranges and limits of detection. Although the present approach requires somewhat long catalytic reaction time (1 h), the Cu-CD/chitosan film composite circumvents the problem of particle aggregation occurred in most particle-based sensing platforms. The Cu-CD/chitosan film was applied to analyze diluted human serum spiked with glucose.

## Supplementary Information

Below is the link to the electronic supplementary material.

Supplementary file1 (DOCX 793 KB)
