# Supplementary material for: Synthesis of Cu-doped carbon dot/chitosan film composite as a catalyst for the colorimetric detection of hydrogen peroxide and glucose
Source: Mikrochim Acta. 2022 Jul 19;189(8):284. doi: 10.1007/s00604-022-05386-3 (PMC9293876; doi:10.1007/s00604-022-05386-3)
Supplement: Supplementary file 1 — Supplementary file1 (DOCX 793 KB) [file 604_2022_5386_MOESM1_ESM.docx]

**Electronic Supplementary Material**

**Synthesis of Cu-doped carbon dots/chitosan film composite as a catalyst for the colorimetric detection of hydrogen peroxide and glucose**

Srikrishna Tummala^a^, Rajkumar Bandi^b^, Yen-Peng-Ho^a*^

^a^ Department of Chemistry, National Dong Hwa University, Hualien 974301, Taiwan

^b^ Institute of Forest Science, Kangwon National University, Chuncheon 23431, Republic of Korea

^*^ Corresponding author: ypho@gms.ndhu.edu.tw

**Synthesis of Cu-CDs**

Cu-CDs were prepared by a simple one-step microwave-assisted approach. In brief, citric acid (300 mg), urea (500 mg), and CuCl_2_.2H_2_O (200 mg) were dissolved in 10 mL of DI water. A beaker containing the solution was placed at the center of a domestic microwave oven (Panasonic NN-ST25JW) and the reaction was proceeded under irradiation at 700 W for 3 min. After the reaction, the solution turned to a dark brown solid. The solid substance was mixed with 20 mL of DI water and centrifuged at 10000 rpm for 15 min to remove insoluble particles. The supernatant was dialyzed in a dialysis tubing (MWCO=1000 Da) for 24 h. The resultant Cu-CDs solution was freeze-dried and stored at 4°C for further use.

**Methods for the detection of H_2_O_2_ and glucose**

Various techniques have been reported for the detection of H_2_O_2_ and glucose based on electrochemical [1-5], chemiluminescence [6,7], fluorescence [8,9], and colorimetric principles [10,11].

**Characterization of Cu-CDs and Cu-CDs/chitosan films**

The morphology of the as-prepared Cu-CDs was investigated by using TEM. The TEM image of Cu-CDs shown in Figure S3a suggests that the particles are mostly in a spherical shape and well dispersed. The Cu-CDs have a narrow size distribution between 1.2 nm to 4 nm with an average size of 2.25 nm (±0.65). The X-ray diffraction (XRD) pattern of Cu-CDs is displayed in Figure 3b. The peak at 27.4° (002) indicates a graphitization structure and a small size of the CDs. FT-IR was used to investigate the functional groups on the surface of Cu-CDs. As shown in Figure 3c, strong absorption peaks at 3446, 3348, and 3186 cm^-1^ are ascribed to O-H and N-H stretching vibrations. The peaks at 2820 and 2780 cm^-1^ are attributed to the C-H stretching. The peaks at 1724, 1630, 1388, and 1245 cm^-1^ correspond to C=O, N-H (amine), C-N, and C-O stretching vibrations, respectively. In addition, the peaks from 900 to 1100 cm^-1^ are assigned to N-Cu-N stretching vibrations [12], suggesting a successful doping of Cu into the CDs framework.

The thickness and morphology of Cu-CDs/chitosan films were measured by using FE-SEM. Figure 3d illustrates the cross-sectional image of the Cu-CDs/chitosan film, which has a thickness of 13.48 ± 4.39 µm (three measurements). The surface morphology is smooth without any large pores as shown in Figure 3e. The surface functionalization of chitosan and Cu-CDs/chitosan films was investigated by using FT-IR spectroscopy. The FT-IR spectrum of chitosan (Figure 3f**)** reveals absorption bands at 3440 cm^-1^ (N-H and O-H stretching), 2930/2880 cm^-1^ (C-H stretching), 1646 cm^-1^ (C=O stretching), 1440 cm^-1^ (C-N stretching), 1358/1150 cm^-1^ (CH_3_ and C-O-C bending (glycosidic linkage)), and 900-1100 cm^-1^ (polysaccharide). The peak broadening at 3400 cm^-1^ on the spectrum of the Cu-CDs/chitosan film may be due to the hydrogen bonding between OH and NH groups of Cu-CDs and chitosan. Comparing the FTIR spectra of the chitosan with the Cu-CDs/chitosan film, we observed a new peak at 1559 cm^-1^ in the spectrum of Cu-CDs/chitosan film. The peak corresponds to the stretching vibration of C=N which is a new bond formed through the crosslinking reaction of chitosan (amine) with glutaraldehyde (aldehyde) [13]. The diameter of the circular Cu-CDs/chitosan film is around 0.6 cm as shown in Figure S4**.**

**Table S1** Comparison of apparent Michaelis constants and maximum reaction rates between previously reported enzymes and the Cu-CDs/chitosan film

| Catalyst | TMB | | H_2_O_2_ | | Reference |
| --- | --- | --- | --- | --- | --- |
|  | K_m_ (mM) | V_m_ (10^-8^Ms^-1^) | K_m_ (mM) | V_m_ (10^-8^Ms^-1^) |  |
| CuNPs | 1.047 | 3.97 | 31.26 | 26.4 | [14] |
| CuNPs/g-C3N4 | 0.389 | 0.584 | 9.27 | 0.84 | [15] |
| HRP | 0.434 | 10 | 3.70 | 8.71 | [16] |
| Cu-CDs /chitosan film | 0.71 | 0.78 | 0.38 | 0.64 | This work |

**Table S2** Detection of spiked glucose content in human serum (n=3)

| Sample | Spiked glucose concentration (µM) | Detected glucose concentration (µM) | Relative standard deviation (±) (n=3) | Recovery (%) |
| --- | --- | --- | --- | --- |
| 1 | 10 | 8.9 | 6.2 | 89 |
| 2 | 20 | 20.1 | 3.3 | 100.4 |
| 3 | 30 | 31.2 | 5.8 | 103.8 |


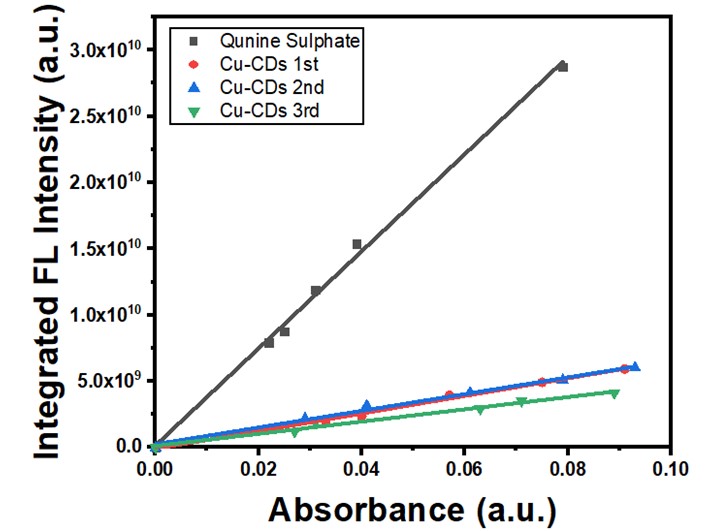


**Figure S1** Calibration curves for quantum yield calculation. The quantum yield (QY) of Cu-CDs was calculated using quinine sulfate in 0.1M H_2_SO_4_ (QY=0.54 at 340 nm) as a reference. The relative QY was calculated by the following equation. Φ_x_= Φ_ST_ (m_x_/m_ST_) (η^2^_x_/ η^2^_ST_) where Φ_x_ and Φ_ST_ are the QYs of the sample and standard (quinine sulfate), respectively. m_x_ and m_ST_ are the slopes of the integrated intensity vs absorption plots for the sample and reference, respectively. η_x_ and η_ST_ are the refractive indexes of the sample and reference, respectively.


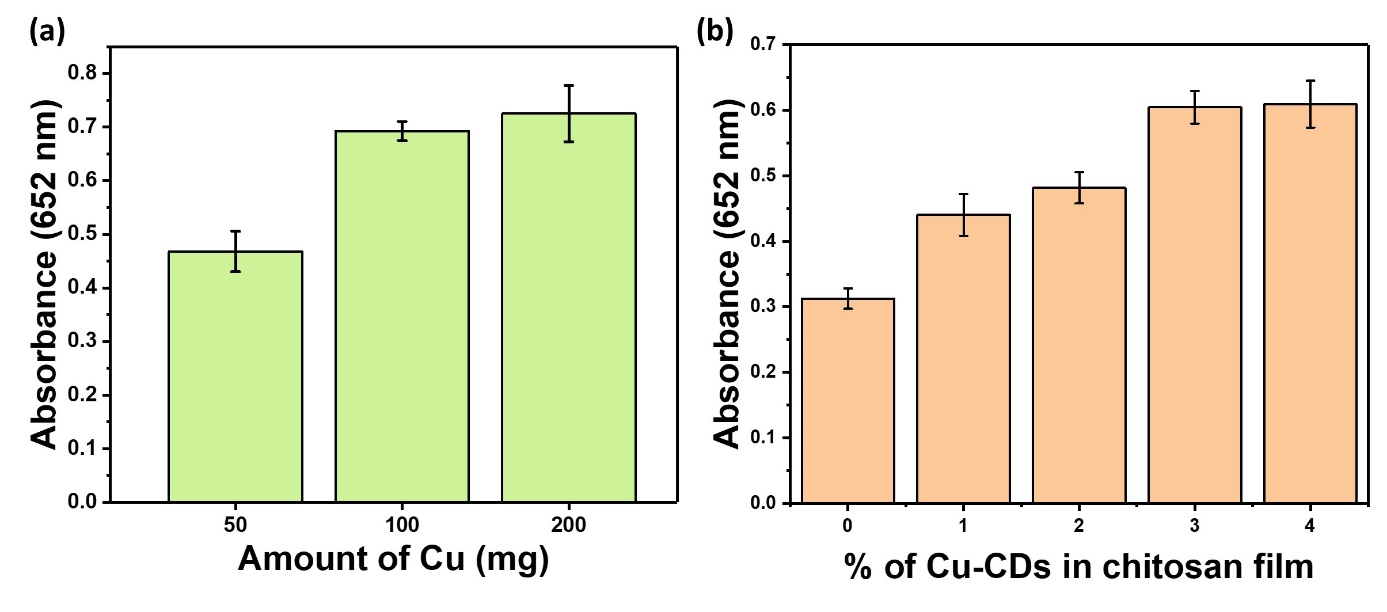


**Figure S2** Optimization of POD activity: a) Optimization of the amount of copper precursor in carbon dots; b) optimization of the percentage of Cu-CDs loaded in chitosan films


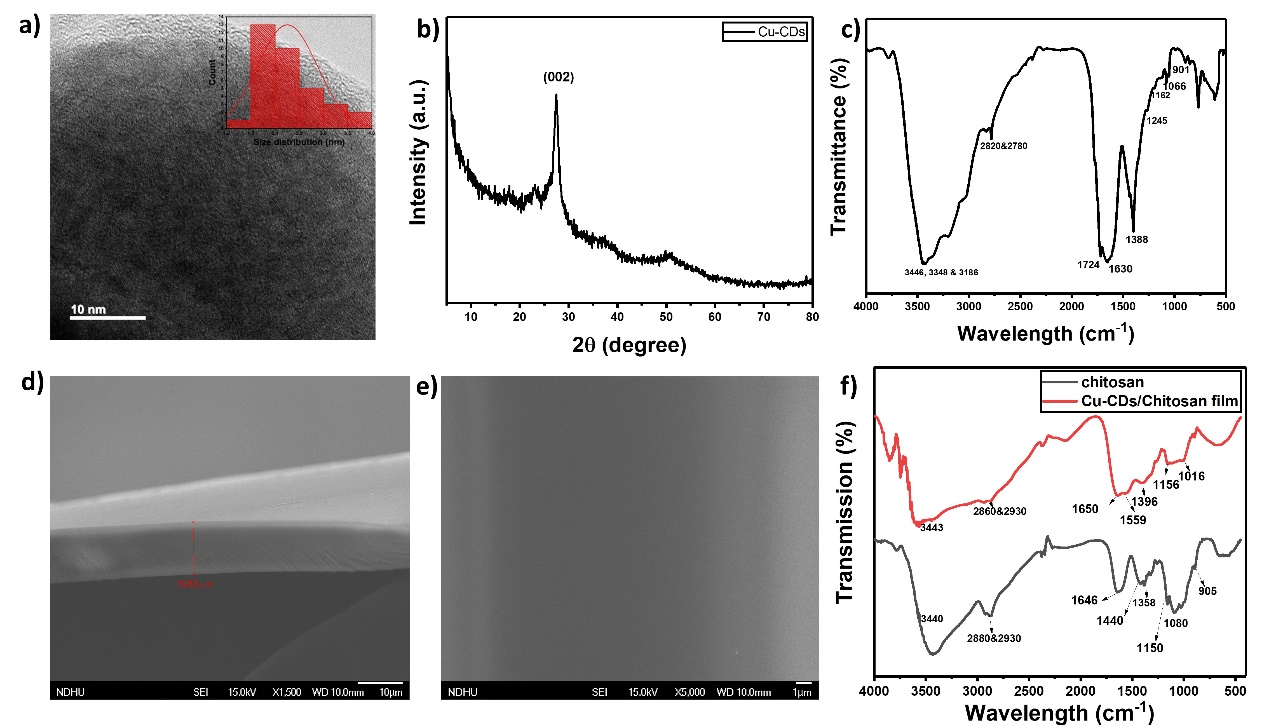


**Figure S3** a) TEM image of Cu-CDs. The inset shows the corresponding particle-size distribution histogram. b) XRD spectrum of Cu-CDs. c) FT-IR spectrum of Cu-CDs. d) SEM cross-sectional image of Cu-CDs/chitosan film with a thickness of 13.48 ± 4.39 µm (n=3). e) SEM surface image of Cu-CDs/chitosan film. f) FT-IR spectra of pure chitosan (black line) and Cu-CDs/chitosan film (red line)

**
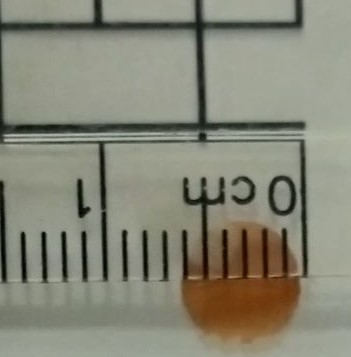
**

**Figure S4** Photograph of Cu-CDs/chitosan film (the diameter is ca. 0.6 cm)

**
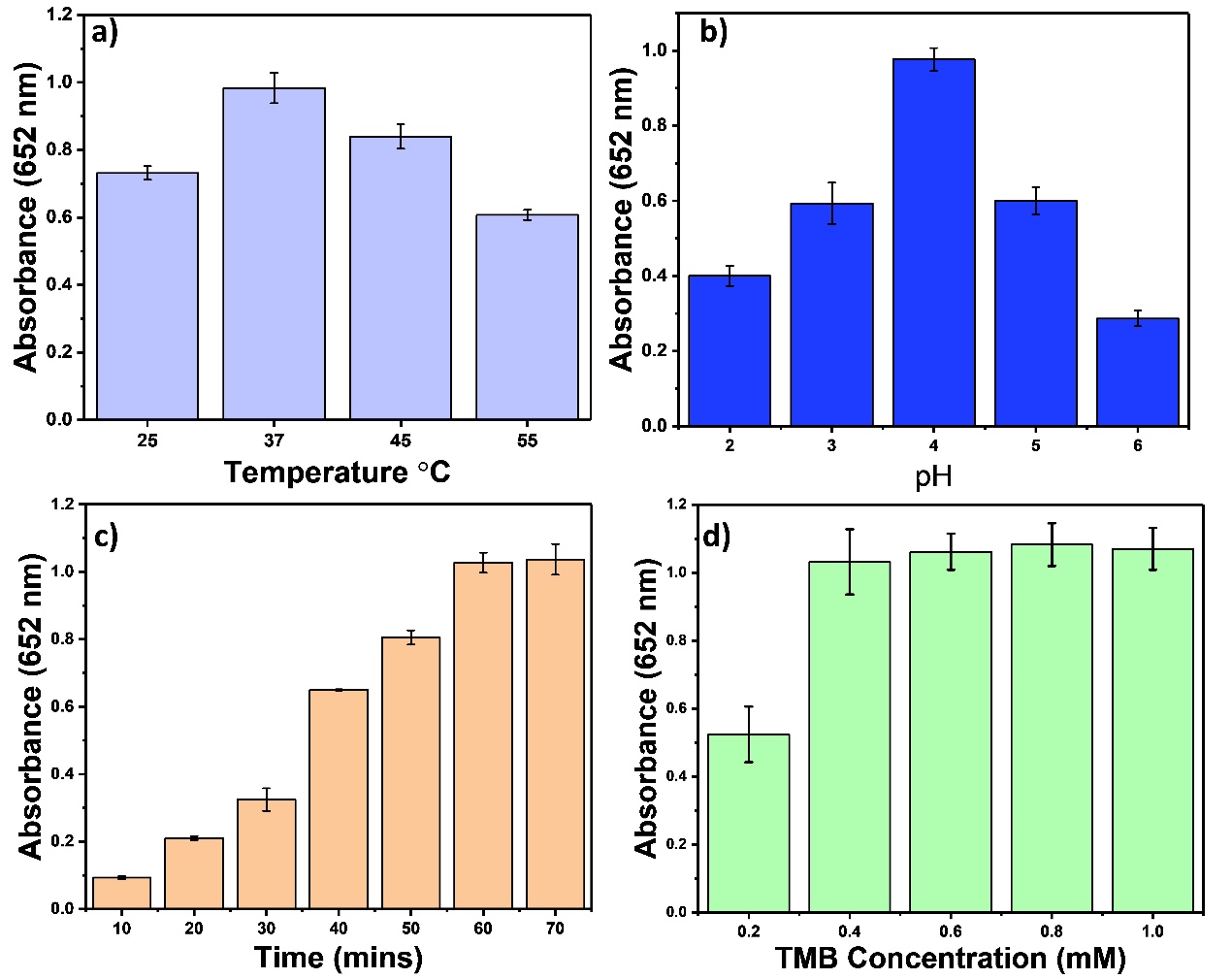
**

**Figure S5** Optimization of peroxidase activity: a) temperature effect, b) effect of solution pH, c) effect of incubation time, and d) effect of TMB substrate concentration

**
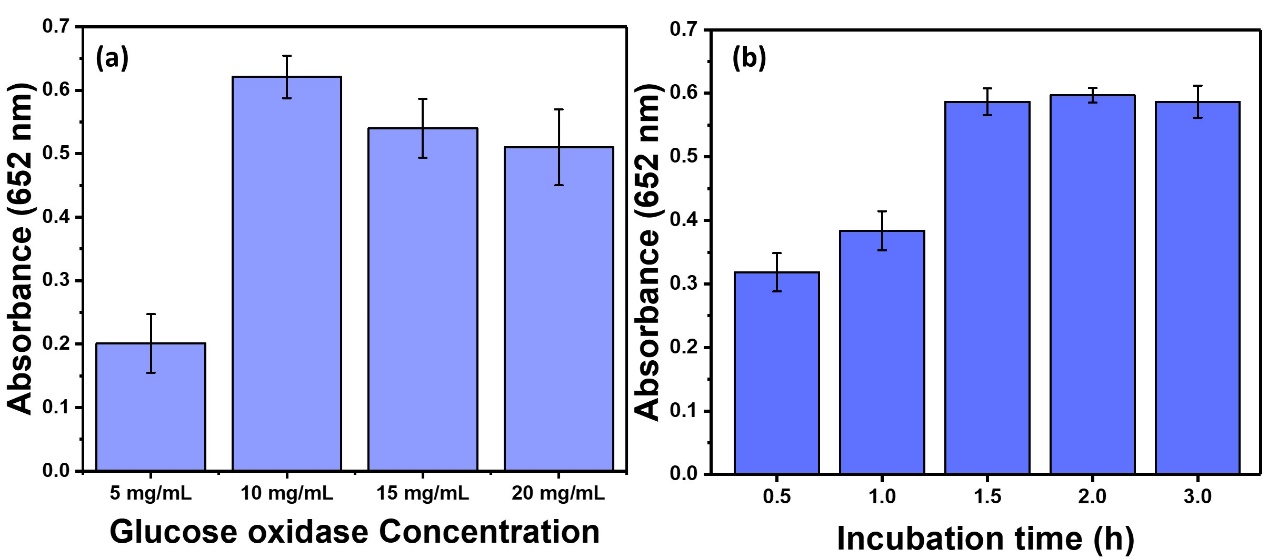
**

**Figure S6** Optimization of glucose oxidase enzyme activity: a) absorbance of oxidized TMB at various concentrations of glucose oxidase; b) absorbance of oxidized TMB at various reaction times

**Figure S7** Selectivity of glucose detection assay when the glucose was mixed with other sugars.

**References**

1. Yuan J, Xu S, Zeng H-Y, Cao X, Dan Pan A, Xiao G-F, Ding P-X (2018) Hydrogen peroxide biosensor based on chitosan/2D layered double hydroxide composite for the determination of H2O2. Bioelectrochemistry 123:94-102. doi:<https://doi.org/10.1016/j.bioelechem.2018.04.009>

2. Gu T, Zhang Y, Deng F, Zhang J, Hasebe Y (2011) Direct electrochemistry of glucose oxidase and biosensing for glucose based on DNA/chitosan film. Journal of Environmental Sciences 23:S66-S69. doi:<https://doi.org/10.1016/S1001-0742(11)61080-2>

3. Shrestha BK, Ahmad R, Mousa HM, Kim I-G, Kim JI, Neupane MP, Park CH, Kim CS (2016) High-performance glucose biosensor based on chitosan-glucose oxidase immobilized polypyrrole/Nafion/functionalized multi-walled carbon nanotubes bio-nanohybrid film. Journal of Colloid and Interface Science 482:39-47. doi:<https://doi.org/10.1016/j.jcis.2016.07.067>

4. Wang Y, Wei W, Liu X, Zeng X (2009) Carbon nanotube/chitosan/gold nanoparticles-based glucose biosensor prepared by a layer-by-layer technique. Materials Science and Engineering: C 29:50-54. doi:<https://doi.org/10.1016/j.msec.2008.05.005>

5. Li J, Yuan R, Chai Y (2011) Simple construction of an enzymatic glucose biosensor based on a nanocomposite film prepared in one step from iron oxide, gold nanoparticles, and chitosan. Microchimica Acta 173:369-374. doi:10.1007/s00604-011-0544-0

6. Deng M, Xu S, Chen F (2014) Enhanced chemiluminescence of the luminol-hydrogen peroxide system by BSA-stabilized Au nanoclusters as a peroxidase mimic and its application. Analytical Methods 6:3117-3123. doi:10.1039/C3AY42135J

7. Chaichi MJ, Ehsani M (2016) A novel glucose sensor based on immobilization of glucose oxidase on the chitosan-coated Fe3O4 nanoparticles and the luminol–H2O2–gold nanoparticle chemiluminescence detection system. Sensors and Actuators B: Chemical 223:713-722. doi:<https://doi.org/10.1016/j.snb.2015.09.125>

8. Wang W-X, Jiang W-L, Liu Y, Li Y, Zhang J, Li C-Y (2020) Near-infrared fluorescence probe with a large stokes shift for visualizing hydrogen peroxide in ulcerative colitis mice. Sensors and Actuators B: Chemical 320:128296. doi:<https://doi.org/10.1016/j.snb.2020.128296>

9. Masteri-Farahani M, Ghorbani F, Mosleh N (2021) Boric acid modified S and N co-doped graphene quantum dots as simple and inexpensive turn-on fluorescent nanosensor for quantification of glucose. Spectrochimica Acta Part A: Molecular and Biomolecular Spectroscopy 245:118892. doi:<https://doi.org/10.1016/j.saa.2020.118892>

10. He S-B, Chen F-Q, Xiu L-F, Peng H-P, Deng H-H, Liu A-L, Chen W, Hong G-L (2020) Highly sensitive colorimetric sensor for detection of iodine ions using carboxylated chitosan–coated palladium nanozyme. Analytical and Bioanalytical Chemistry 412:499-506. doi:10.1007/s00216-019-02270-7

11. Cui Y, Lai X, Liang B, Liang Y, Sun H, Wang L (2020) Polyethyleneimine-Stabilized Platinum Nanoparticles as Peroxidase Mimic for Colorimetric Detection of Glucose. ACS Omega 5:6800-6808. doi:10.1021/acsomega.0c00147

12. Ma Y, Cen Y, Sohail M, Xu G, Wei F, Shi M, Xu X, Song Y, Ma Y, Hu Q (2017) A Ratiometric Fluorescence Universal Platform Based on N, Cu Codoped Carbon Dots to Detect Metabolites Participating in H2O2-Generation Reactions. ACS Applied Materials & Interfaces 9:33011-33019. doi:10.1021/acsami.7b10548

13. Li B, Shan C-L, Zhou Q, Fang Y, Wang Y-L, Xu F, Han L-R, Ibrahim M, Guo L-B, Xie G-L, Sun G-C (2013) Synthesis, Characterization, and Antibacterial Activity of Cross-Linked Chitosan-Glutaraldehyde. Mar Drugs 11:1534-1552

14. Wang N, Li B, Qiao F, Sun J, Fan H, Ai S (2015) Humic acid-assisted synthesis of stable copper nanoparticles as a peroxidase mimetic and their application in glucose detection. Journal of Materials Chemistry B 3:7718-7723. doi:10.1039/C5TB00684H

15. Wang N, Han Z, Fan H, Ai S (2015) Copper nanoparticles modified graphitic carbon nitride nanosheets as a peroxidase mimetic for glucose detection. Rsc Adv 5:91302-91307. doi:10.1039/C5RA18957H

16. Gao L, Zhuang J, Nie L, Zhang J, Zhang Y, Gu N, Wang T, Feng J, Yang D, Perrett S, Yan X (2007) Intrinsic peroxidase-like activity of ferromagnetic nanoparticles. Nature Nanotechnology 2:577-583. doi:10.1038/nnano.2007.260
